# Organizational Culture Patterns and Safety‐Relevant Dimensions in Teaching Hospitals: A Cross‐Sectional Study

**DOI:** 10.1002/hsr2.72779

**Published:** 2026-07-06

**Authors:** Leili Alizamani, Ehsan Mousa‐Farkhani, Mahin Esmaeili‐Darmian, Ali Vafaee‐Najar, Elaheh Hooshmand

**Affiliations:** ^1^ Student Research Committee Mashhad University of Medical Sciences Mashhad Iran; ^2^ Department of Epidemiology, School of Health Mashhad University of Medical Sciences Mashhad Iran; ^3^ Social Determinants of Health Research Center Mashhad University of Medical Sciences Mashhad Iran; ^4^ Department of Management Sciences and Health Economics, School of Health Mashhad University of Medical Sciences Mashhad Iran

**Keywords:** accountability, cross‐sectional study, low‐ and middle‐income countries (LMICs), organizational culture, patient safety, responsibility, teaching hospitals

## Abstract

**Background and Aims:**

Organizational culture is widely recognized as an important determinant of patient safety, yet it remains unclear which cultural dimensions genuinely drive safety‐related outcomes in teaching hospitals, particularly in low‐ and middle‐income country (LMIC) contexts. This study aimed to move beyond organizational ideals by identifying dominant cultural patterns and their potential operational relevance for safety outcomes in Iranian teaching hospitals.

**Methods:**

This cross‐sectional analytical study was conducted among healthcare staff working in teaching hospitals affiliated with Mashhad University of Medical Sciences, Iran. Organizational culture was assessed using a validated multilevel questionnaire measuring teamwork, responsibility, risk‐taking, and accountability across beliefs, values, behaviors, and symbols. Descriptive statistics, group comparisons, correlation analyses, and regression models were used to examine cultural patterns and identify factors associated with accountability and responsibility.

**Results:**

A total of 108 hospital staff participated in the study. Among the four organizational culture dimensions, responsibility showed the highest overall mean score, followed by accountability, while teamwork and risk‐taking had comparatively lower mean scores. Female participants reported significantly higher responsibility scores than male participants (4.17 ± 0.69 vs. 3.85 ± 0.54; *p* = 0.014). Significant differences were observed across staff categories, with managerial staff reporting higher accountability scores (4.20 ± 0.61; *p* = 0.019). Clinical staff reported higher responsibility scores (4.18 ± 0.66; *p* = 0.047), indicating a modest but statistically significant difference compared to other staff groups. Teamwork was strongly correlated with accountability (*r* = 0.696, *p* < 0.01), suggesting a robust positive relationship between collaborative culture and accountability‐related organizational behaviors.

**Conclusion:**

Teaching hospitals in Iran exhibit a predominantly conservative organizational culture that prioritizes stability and compliance over adaptability and innovation. While responsibility and accountability represent important strengths, strengthening teamwork and collaborative practices may enhance the practical translation of organizational values into safety‐related outcomes. These findings offer relevant insights for teaching hospitals in Iran and other LMIC settings seeking to balance accountability with adaptability in pursuit of safer and more resilient health systems.

## 1 Introduction

Organizational culture is increasingly recognized as a critical determinant of health system performance and human resources for health (HRH) outcomes, shaping how healthcare professionals interact, make decisions, and respond to everyday challenges [1].

Poor patient safety culture has been consistently linked to adverse events, increased medical errors, prolonged hospital stays, and higher healthcare costs [2, 3]. In teaching hospitals, where clinical complexity and workforce training intersect, weak safety culture may not only compromise current patient outcomes but also shape unsafe practices among future healthcare professionals. Therefore, strengthening organizational culture is not only a managerial concern but a critical public health priority [4].

Organizational culture plays a central role in shaping safety‐related behaviors, communication patterns, and decision‐making processes within healthcare settings. A positive safety culture has been associated with reduced medical errors, improved reporting of adverse events, and enhanced patient outcomes. Conversely, fragmented or hierarchical cultures may limit open communication and hinder effective safety practices [5]. Therefore, understanding which cultural dimensions are most closely linked to safety‐related behaviors is essential for designing targeted interventions in teaching hospitals.

In teaching hospitals—where clinical service delivery intersects with workforce training—the cultural environment plays a particularly influential role, affecting not only patient care but also the development of future health professionals [6]. While values such as responsibility and accountability are widely promoted as cornerstones of effective healthcare organizations, their actual contribution to measurable safety‐related outcomes is often assumed rather than empirically clarified [7].

Evidence from high‐income countries suggests that balanced organizational cultures—integrating teamwork, accountability, and adaptability—are associated with improved patient safety and institutional resilience [8]. In contrast, health systems in low‐ and middle‐income countries (LMICs) frequently operate under resource constraints, workforce shortages, and high service demands—conditions that tend to reinforce conservative cultural orientations emphasizing compliance and stability over innovation [9]. Although such cultures may support order and control, it remains unclear which organizational attributes genuinely function as effective levers to contribute to safer care environments.

In Iran, teaching hospitals form the backbone of healthcare service provision and medical education, delivering a substantial proportion of inpatient care nationwide through their integration within the Ministry of Health and Medical Education system [10]. These hospitals face the dual pressure of maintaining service quality while training a diverse and expanding workforce. Previous studies have suggested strong orientations toward responsibility and accountability within Iranian hospital cultures [11, 12]; Many of these studies have relied on cross‐sectional designs, self‐reported measures, and primarily descriptive analytical approaches, which limit causal inference and reduce the ability to establish robust relationships between organizational culture dimensions and safety‐related outcomes [7]. In contrast to previous studies, the present research applies a multilevel cultural framework that simultaneously examines beliefs, values, behaviors, and symbols. In addition, it uses analytical methods beyond simple descriptive comparisons, including correlation and regression analyses, to better identify relationships among cultural dimensions and their potential operational relevance.

Mashhad University of Medical Sciences (MUMS) is one of the largest medical universities in Iran, operating an extensive network of affiliated teaching hospitals and urban and rural healthcare centers that collectively function as major referral hubs for specialized care. This integrated structure supports both healthcare service delivery and medical education, exposing a heterogeneous patient population and reflecting organizational complexities commonly observed in LMIC health systems [9, 10, 11, 12, 13]. MUMS teaching hospitals operate within a complex organizational structure characterized by high patient volumes, diverse clinical specialties, multiple professional groups, and simultaneous responsibilities for service delivery and medical education. These features create layered decision‐making processes and interdependent workflows that are representative of teaching hospital environments in LMIC contexts.

By applying a validated multilevel cultural framework encompassing beliefs, values, behaviors, and symbols, the present study moves beyond organizational ideals to examine patterns of organizational culture and identify relationships among key cultural dimensions with potential relevance to safety‐related practices in teaching hospitals within an LMIC context. Therefore, the objective of this study was to examine patterns of organizational culture and identify which cultural dimensions are most strongly associated with key organizational attributes in teaching hospitals within an LMIC context.

The findings aim to help managers and policymakers distinguish between culturally dominant attributes and those with genuine practical leverage for improving safety performance in resource‐constrained health systems.

## 2 Materials and Methods

This cross‐sectional analytical study was conducted in 2024 at teaching hospitals affiliated with Mashhad University of Medical Sciences (MUMS), one of the largest academic medical centers in Iran. As a major referral hub in the northeast of the country, MUMS teaching hospitals deliver a substantial volume of inpatient and outpatient services while simultaneously functioning as core training sites for medical and health sciences students. These hospitals, therefore, reflect the organizational complexity typical of teaching hospitals in low‐ and middle‐income countries (LMICs), where service provision, workforce training, and resource constraints coexist.

The study received ethical approval from the Institutional Review Board of Mashhad University of Medical Sciences (IR.MUMS.REC.1403.084). Participation was voluntary, written informed consent was obtained from all respondents, and anonymity and confidentiality were strictly maintained throughout the study.

The study was designed to move beyond descriptive assessments of organizational culture and to identify which organizational factors are most strongly associated with safety‐related outcomes in teaching hospitals operating within an LMIC context.

The study population included healthcare professionals working in MUMS teaching hospitals, encompassing physicians, nurses, and allied health staff directly involved in patient care. Due to limitations in access to centralized workforce data, an exact total population size could not be determined; however, efforts were made to ensure adequate representation across professional groups.

A stratified sampling approach was used to ensure representation across key professional groups. First, the study population was divided into strata based on professional role (physicians, nurses, and allied health staff). Within each stratum, participants were proportionally selected based on availability during the data collection period. Efforts were made to include participants from different hospital units to capture variability in organizational culture. A minimum of 1 year of work experience was required to ensure that participants had sufficient exposure to the organizational environment and culture of the hospital. This criterion increases the likelihood that responses reflect stable perceptions rather than initial impressions.

Staff with exclusively administrative roles were excluded to maintain focus on personnel directly involved in patient care processes, where organizational culture is more directly linked to safety‐related behaviors. Including administrative staff could have introduced additional heterogeneity not directly related to clinical safety dynamics. Based on recommendations for regression analysis, a minimum sample size of 10–15 observations per independent variable was considered adequate.

Organizational culture was assessed using a validated questionnaire originally developed for Iranian healthcare settings [14], making it particularly suitable for examining organizational dynamics in teaching hospitals within an LMIC environment. In the present study, the instrument underwent a rigorous validation process. Content validity was confirmed by an expert panel, yielding a Content Validity Ratio (CVR) of 0.75 and a Content Validity Index (CVI) of 0.96. Internal consistency reliability was high, with a Cronbach's alpha of 0.97.

The questionnaire consisted of two sections [1]: demographic and occupational characteristics (11 items), and [2] organizational culture assessment (48 items). The applied framework conceptualizes organizational culture across four layers—beliefs, values, behaviors, and symbols—allowing for a comprehensive assessment of both underlying assumptions and observable practices. A sample of the questionnaire has been provided as supporting material to facilitate replication and further research.

The organizational culture section evaluated four key components—teamwork, responsibility, risk‐taking, and accountability—across four cultural layers (beliefs, values, behaviors, and symbols), forming a multilevel cultural framework. This structure enabled the simultaneous examination of culturally dominant organizational ideals and operational behaviors that may differentially translate into observable safety‐related outcomes in teaching hospital settings. Responses were recorded on a six‐point Likert scale ranging from 1 (never) to 6 (very high).

While the instrument demonstrated strong reliability and content validity, it relies on self‐reported perceptions, which may be influenced by social desirability bias. Additionally, the cross‐sectional nature of measurement limits the ability to capture dynamic changes in organizational culture over time.

Following approval from the Research Ethics Committee of Mashhad University of Medical Sciences. Participants were informed of the study objectives, assured of anonymity and confidentiality, and provided written informed consent prior to participation. Completed questionnaires were reviewed for completeness before data entry.

Data were collected using self‐administered paper‐based questionnaires distributed during working hours. Participation was voluntary and anonymous. To minimize potential recruitment bias, participants were informed that responses would remain confidential and would not be shared with supervisors or hospital management. No identifying information was collected.

Completed questionnaires were checked for completeness and then entered into the database. Data entry was reviewed for accuracy through random checks of selected questionnaires.

All data were stored in password‐protected files accessible only to the research team. Physical questionnaires were securely stored and later destroyed after data entry.

Data were coded and analyzed using SPSS version 22 and Microsoft Excel 2013. Descriptive statistics, including frequencies, means, and standard deviations, were used to summarize participant characteristics and organizational culture scores.

Inferential analyses were conducted to examine differences and relationships among organizational culture dimensions and demographic variables. Independent‐samples *t*‐tests were used to compare mean scores between two groups, such as male and female participants. One‐way analysis of variance (ANOVA) was applied to assess differences in organizational culture dimensions across multiple staff categories (clinical, administrative, and managerial), with post‐hoc tests performed where significant differences were identified.

Pearson correlation coefficients were used to examine the strength and direction of relationships among the four organizational culture dimensions (teamwork, responsibility, risk‐taking, and accountability). Linear regression models were employed to identify predictors of accountability and responsibility, which were treated as dependent variables. Independent variables included gender, years of service, staff category, and education level.

Rather than focusing solely on statistical significance, the analytical strategy aimed to distinguish between organizational factors with symbolic prominence and those demonstrating measurable associations with safety‐related outcomes. This approach was intended to highlight organizational levers with practical relevance for safety improvement in teaching hospitals operating under resource constraints.

All statistical tests were two‐sided, and a significance level of *p* < 0.05 was considered statistically significant. Effect sizes and descriptive statistics were reported alongside *p*‐values to provide a more comprehensive interpretation of the results.

## 3 Results

A total of 120 questionnaires were distributed, of which 108 were returned, yielding a response rate of 90%. All returned questionnaires were reviewed for completeness, and no major missing data were identified; therefore, all 108 responses were included in the final analysis.

As shown in Table 1, the majority of participants were female (73/108, 67.6%) and married (82/108, 75.9%). Most respondents held a bachelor's degree (61/108, 56.5%) and had 5–10 years of work experience (36/108, 33.3%). Nearly one‐third of participants (32/108, 29.6%) reported previous managerial experience, reflecting the presence of diverse professional roles within the teaching hospital setting.

**Table 1:** Demographic characteristics of participants (*N* = 108).

Characteristic	Category	Frequency	%
Employment status	Formal	47	43.5
	Contractual	31	28.7
	Temporary	22	20.4
	Other	8	7.4
Gender	Male	35	32.4
	Female	73	67.6
Marital status	Married	82	75.9
	Single	26	24.1
Education	Bachelor	61	56.5
	Master's	37	34.3
	PhD	7	6.5
Years of service	< 5	21	19.4
	5–10	36	33.3
	10–15	26	24.1
	≥ 15	25	23.1

**Figure 1:**
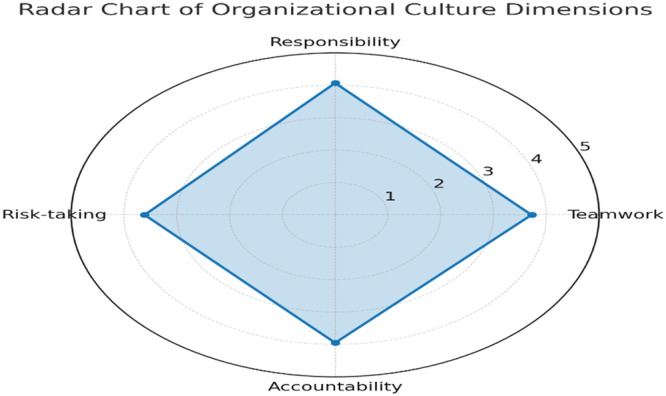
Distribution of organizational culture dimensions (teamwork, responsibility, risk‐taking, and accountability) among hospital staff, presented as mean ± standard deviation (SD) scores.

The overall distribution of organizational culture dimensions is illustrated in Figure 1. Responsibility showed the highest mean scores across dimensions (*M* = 4.13 ± 0.81), followed by accountability (*M* = 3.99 ± 0.77), while teamwork and risk‐taking had comparatively lower mean values.

A more detailed examination of these dimensions across cultural layers is presented in Table 2. Responsibility showed the highest mean scores at the levels of beliefs and behaviors, while accountability was most prominent at the symbolic level. In contrast, risk‐taking consistently demonstrated the lowest scores across all layers, indicating limited organizational tolerance for uncertainty and innovation.

**Table 2:** Cultural components across belief systems.

Component	Beliefs (M ± SD)	Values (M ± SD)	Behaviors (M ± SD)	Symbols (M ± SD)
Teamwork	3.65 ± 0.88	3.88 ± 0.91	3.63 ± 0.95	3.62 ± 0.93
Responsibility	4.13 ± 0.81	3.96 ± 0.68	4.13 ± 1.45	4.05 ± 0.81
Risk‐taking	3.53 ± 0.72	3.66 ± 0.71	3.56 ± 0.73	3.63 ± 1.21
Accountability	3.65 ± 0.81	3.75 ± 0.83	3.99 ± 0.77	4.10 ± 0.72

Gender‐based comparisons of organizational culture dimensions are summarized in Table 3. Female participants reported higher responsibility scores (4.17 ± 0.69 vs. 3.85 ± 0.54; *p* = 0.014) compared to male participants (*p* = 0.014). No statistically significant gender differences were observed for teamwork, risk‐taking, or accountability.

**Table 3:** Organizational culture dimensions by gender.

Dimension	Male (M ± SD)	Female (M ± SD)	*t*‐value	*p* value
Teamwork	3.70 ± 0.47	3.69 ± 0.90	0.04	0.97
Responsibility	3.85 ± 0.54	4.17 ± 0.69	−2.49	0.014*
Risk‐taking	3.57 ± 0.30	3.61 ± 0.71	−0.23	0.82
Accountability	3.82 ± 0.47	3.90 ± 0.66	−0.48	0.63

*Note:* Statistical significance was considered at *p* < 0.05.

Table 3 presents the organizational culture dimensions by gender. Female participants reported significantly higher responsibility (*p* = 0.014).

**Figure 2:**
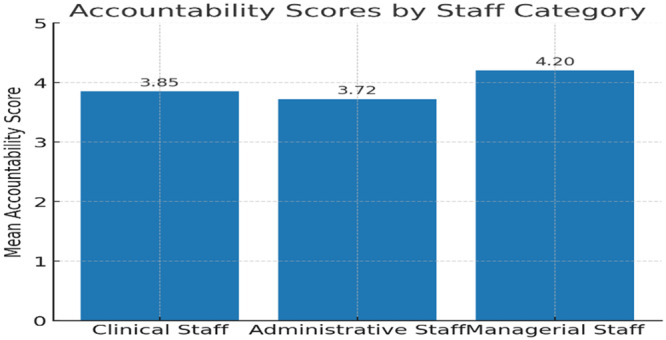
Comparison of organizational culture dimensions (teamwork, responsibility, risk‐taking, and accountability) across staff categories (clinical, administrative, and managerial), presented as mean scores.

Differences in organizational culture dimensions across staff categories are presented in Table 4 and illustrated in Figure 2. Managerial staff demonstrated significantly higher accountability scores compared to clinical and administrative staff (*p* = 0.019). In contrast, clinical staff reported the highest responsibility scores among the three groups (*p* = 0.047). No statistically significant differences were observed across staff categories for teamwork or risk‐taking.

**Table 4:** Organizational culture dimensions by staff category (ANOVA).

Dimension	Clinical (M ± SD)	Administrative (M ± SD)	Managerial (M ± SD)	*F*‐value	*p* value
Teamwork	3.75 ± 0.81	3.65 ± 0.79	3.78 ± 0.84	0.28	0.76
Responsibility	4.18 ± 0.66	3.90 ± 0.57	4.05 ± 0.62	3.15	0.047*
Risk‐taking	3.59 ± 0.70	3.55 ± 0.66	3.68 ± 0.71	0.41	0.66
Accountability	3.85 ± 0.54	3.72 ± 0.49	4.20 ± 0.61	4.12	0.019*

* *p* < 0.05.

The interrelationships among organizational culture dimensions are shown in Table 5. All four dimensions were positively and significantly correlated. Statistical analyses were conducted using SPSS version 22. All tests were two‐sided, and a significance level of *p* < 0.05 was considered statistically significant.

**Table 5:** Correlation matrix of cultural components.

Component	Responsibility	Risk‐taking	Teamwork	Accountability
Responsibility	1	0.233*	0.383**	0.511**
Risk‐taking	0.233*	1	0.543**	0.430**
Teamwork	0.383**	0.543**	1	0.696**
Accountability	0.511**	0.430**	0.696**	1

* *p* < 0.05;

** *p* < 0.01.

The strongest association was observed between teamwork and accountability (*r* = 0.696), followed by the association between responsibility and accountability (*r* = 0.511), indicating substantial interdependence among cultural components. Linear regression analysis was conducted to identify predictors of accountability and responsibility. For accountability, years of service showed a positive association (*β* = 0.21, *p* = 0.064), indicating a trend toward higher accountability with longer tenure. Staff category was also associated with accountability, with managerial roles reporting higher scores (*β* = 0.27, *p* = 0.031).

For responsibility, gender was a significant predictor (*β* = 0.24, *p* = 0.022), with female staff reporting higher responsibility scores. No significant associations were observed for education level (*p* > 0.05).

The regression models explained a modest proportion of variance in accountability (*R* ^2^ = 0.18) and responsibility (*R* ^2^ = 0.15), suggesting that additional organizational and contextual factors may contribute to variations in these cultural dimensions.

Taken together, the findings indicate that responsibility and accountability function as dominant organizational ideals in teaching hospitals within this LMIC context. However, teamwork—despite its comparatively lower mean scores—demonstrated strong relational connectivity with accountability, suggesting greater operational relevance beyond symbolic prominence. This distinction highlights that not all culturally dominant dimensions necessarily act as primary drivers of safety‐related outcomes.

Although this study did not directly measure objective patient safety outcomes, the observed relationships among cultural dimensions—particularly the strong association between teamwork and accountability—suggest potential pathways through which organizational culture may influence safety‐related behaviors. In teaching hospitals, where coordination and communication are essential, strengthening teamwork may enhance the practical implementation of accountability mechanisms and contribute to safer care delivery.

## 4 Discussion

This study demonstrated that responsibility and accountability represent the dominant cultural strengths in Iranian teaching hospitals, whereas teamwork and risk‐taking are comparatively less emphasized. Stratified analyses revealed meaningful role‐based differences: managerial staff reported stronger accountability, while responsibility was most prominent among clinical employees. Collectively, these patterns point to a conservative organizational culture in which stability, ethical commitment, and compliance are prioritized over innovation and adaptability, which has been associated with variations in patient safety performance in healthcare settings [15, 16].

The findings also suggest that demographic and professional characteristics influence organizational culture perceptions. For instance, longer years of service were associated with higher accountability, which may reflect increased familiarity with organizational norms and expectations. Similarly, variations across staff categories highlight how hierarchical roles shape cultural perceptions within hospital settings. Differences related to employment status and educational level, although less pronounced, further indicate that organizational culture is experienced differently across subgroups.

Regression findings further indicated that demographic and professional characteristics matter, with female staff reporting higher responsibility and longer tenure being associated with stronger accountability.

A key contribution of this study lies in distinguishing culturally dominant ideals from dimensions with potential operational relevance for safety outcomes. Although teamwork scored lower than responsibility and accountability, its strong relational connectivity with accountability suggests that collaborative practices may play a critical enabling role in translating formal accountability into everyday safety‐related behaviors. Although this study did not directly measure objective patient safety outcomes, the findings provide insight into how organizational culture may influence safety‐related practices. In particular, the strong association between teamwork and accountability suggests that collaborative environments may facilitate the implementation of accountability mechanisms, which are essential for maintaining patient safety in complex clinical settings.

This finding is particularly salient in teaching hospitals, where patient care, supervision, and training are inherently interdependent and require effective coordination across professional boundaries. This finding is consistent with previous research highlighting the central role of teamwork and communication in shaping patient safety culture and improving coordination in hospital settings [17].

One of the major strengths of this study is the application of a multilevel cultural framework encompassing beliefs, values, behaviors, and symbols, allowing for a more nuanced assessment of organizational culture than single‐layer approaches. The use of a validated instrument with excellent internal consistency (Cronbach's *α* = 0.97) and strong content validity indices further strengthens confidence in the findings. Stratified sampling also enhanced representativeness across different hospital roles.

Several limitations should be acknowledged. The cross‐sectional design precludes causal inference. Self‐reported measures may be subject to social desirability or recall bias. Although stratified sampling was employed, analyses did not incorporate weighted cluster modeling, which may have introduced some imprecision. While the questionnaire demonstrated strong internal consistency and content validity, it is important to note that it relies on self‐reported perceptions, which may be subject to social desirability bias. In addition, although the instrument captures multiple cultural layers, it may not fully reflect dynamic or context‐specific variations in organizational culture. Future studies may benefit from combining such tools with observational or qualitative approaches to achieve a more comprehensive understanding. It should also be noted that some professional groups may have been under‐represented in the sample, particularly due to differences in availability during the data collection period. This may limit the generalizability of findings across all human resources for health (HRH) categories.

Finally, the study was conducted within a single academic institution, limiting generalizability; nevertheless, the findings remain highly relevant to similar teaching hospitals in low‐ and middle‐income countries (LMICs).

When situated within the broader literature, the findings align with organizational culture patterns reported in other LMIC settings. A study from Ethiopia has identified dominant hierarchical and conservative cultures in hospitals, characterized by limited innovation and flexibility despite staff preferences for more collaborative and adaptive environments [1]. Iranian study has similarly emphasized internal cohesion, responsibility, and control as defining cultural traits [2]. In contrast, evidence from high‐income countries suggests more balanced cultural profiles that integrate teamwork, adaptability, and distributed leadership alongside accountability [3]. Similar patterns have been reported in other healthcare systems, where organizational context and leadership structures play a key role in shaping safety culture development over time [18].

Gender‐related differences observed in this study are also consistent with findings from LMIC contexts. A scoping review of fragile and conflict‐affected settings highlighted how professional norms and care burdens shape women's experiences within the health workforce [19]. The “double burden” phenomenon—where women combine formal employment with disproportionate unpaid domestic responsibilities—may help explain higher perceived responsibility among female staff and is more pronounced in LMICs [20]. Leadership literature further suggests that managerial roles are commonly associated with accountability; however, in high‐income settings this is often coupled with innovation and autonomy, a pattern less evident in the present study [8].

These findings underscore the importance of distinguishing between culturally dominant attributes and those with operational relevance. While responsibility and accountability appear prominent, their effectiveness in improving safety outcomes may depend on complementary dimensions such as teamwork and communication. This highlights the need for balanced cultural development rather than reinforcing already dominant attributes. This is consistent with recent reviews emphasizing the importance of balanced cultural development in improving safety and quality outcomes in healthcare organizations [1, 15]. These observations are supported by recent evidence demonstrating that improvements in organizational culture can contribute to enhanced safety‐related outcomes [1, 15].

From a policy perspective, these findings underscore the importance of strengthening teamwork and adaptability in Iranian teaching hospitals without undermining the positive foundations of responsibility and accountability. Leadership development programs should promote participatory decision‐making, shared accountability, and managed risk‐taking, fostering a culture of learning and innovation rather than reinforcing purely hierarchical control mechanisms [7, 21]. Gender‐sensitive human resources for health (HRH) policies are also needed to recognize and support women's contributions, including equitable career progression and leadership opportunities. More broadly, embedding multilevel cultural diagnostics into HRH planning may help identify conservative biases and guide interventions that foster innovation and collaboration [20].

This study contributes to the international evidence base by illustrating how organizational culture varies across professional roles, gender, and experience in a middle‐income teaching hospital context. Iranian teaching hospitals appear to prioritize stability over adaptability, reflecting patterns observed across many LMICs and diverging from the more balanced cultural configurations of high‐income systems. By strengthening teamwork and innovation while preserving accountability, teaching hospitals in Iran and comparable settings can move toward more resilient, responsive, and equitable health systems. From an Asia–Pacific perspective, where many health systems face similar structural constraints and workforce challenges, these findings offer insights with relevance beyond the Iranian context.

## 5 Conclusion

This study demonstrates that teaching hospitals in Iran are characterized by a predominantly conservative organizational culture, in which responsibility and accountability represent core strengths, while teamwork and risk‐taking remain comparatively underdeveloped. Although this cultural configuration supports stability and compliance, it may limit adaptability and innovation—capacities that are increasingly essential in complex and rapidly evolving healthcare systems. Strengthening collaborative practices and encouraging shared accountability may therefore enhance the translation of formal organizational values into effective safety‐related behaviors.

These findings have relevance beyond the Iranian context, as many teaching hospitals in low‐ and middle‐income countries exhibit similar cultural patterns shaped by resource constraints and hierarchical structures. From a policy and management perspective, aligning organizational culture with human resources for health strategies is critical to sustaining workforce engagement and improving care quality. Building cultures that balance accountability with adaptability may help teaching hospitals move toward more resilient, responsive, and equitable health systems.

## Author Contributions

**Leili Alizamani:** conceptualization, data collection, and drafting of the manuscript. **Ehsan Mousa‐Farkhani:** methodology, statistical analysis, and data interpretation. **Mahin Esmaeili‐Darmian:** literature review, critical revision, and visualization. **Ali Vafaee‐Najar:** supervision, validation, administrative and technical support. **Elaheh Hooshmand:** project administration, study design, and final approval of the version to be published.

## Ethics Statement

This research is approved by the Ethics Committee of Mashhad University of Medical Sciences (IR. MUMS.REC.1403.084).

## Conflicts of Interest

The authors declare no conflicts of interest.

## Transparency Statement

All methods and materials used in this research are described in detail in the methods section of this article to allow for replication. Elaheh Hooshmand affirms that this manuscript is an honest, accurate, and transparent account of the study being reported.

## Supporting information


Supporting File

## Data Availability

The data that support the findings of this study are available from the corresponding author upon reasonable request.
