# Supplementary material for: Organizational Culture Patterns and Safety‐Relevant Dimensions in Teaching Hospitals: A Cross‐Sectional Study
Source: Health Sci Rep. 2026 Jul 6;9(7):e72779. doi: 10.1002/hsr2.72779 (PMC13338568; doi:10.1002/hsr2.72779)
Supplement: Supplementary file 1 — Supporting File [file HSR2-9-e72779-s001.docx]

**Supplementary File 1
Structure and Sample Items of the Organizational Culture Questionnaire**

The organizational culture questionnaire used in this study was adapted from the instrument developed by Mosadeghrad et al. for Iranian healthcare organizations. The tool was specifically designed for hospital settings and evaluates organizational culture across two major dimensions:

1. Internal Coherence
2. External Adaptation

These dimensions include four organizational culture components:

- Teamwork
- Accountability
- Risk-taking
- Responsiveness

The questionnaire contains 48 items distributed across four cultural levels:

- Beliefs
- Values
- Behaviors
- Symbols

Each component included 12 items, with three items representing each cultural level.

Responses were scored using a 5-point Likert scale ranging from very low to very high. Higher scores indicated stronger organizational culture attributes.

Examples of questionnaire items include:

Teamwork

- “Knowledge and experience sharing among staff is encouraged.”
- “Team-based performance is valued within the hospital.”

Accountability

- “Clear job responsibilities are defined for staff.”
- “Employees are encouraged to take responsibility for their duties.”

Risk-taking

- “Managers support innovative ideas and creative solutions.”
- “Change is viewed as an opportunity for improvement.”

Responsiveness

- “Patient satisfaction is considered a major organizational priority.”
- “Hospital staff are responsive to patients’ concerns and expectations.”

The questionnaire was previously developed and psychometrically evaluated in Iranian hospital settings by Mosadeghrad et al.

Reference:
Mosadeghrad AM, Parsaeian M, Hedayati SP. Organizational culture of hospitals affiliated to Tehran University of Medical Sciences. Payesh. 2023;22(4):373–385.
